# Trauma, Hyperarousal, and Sleep: Neurobehavioral Pathways in Affective Disorders

**DOI:** 10.3390/bs16101764

**Published:** 2026-09-28

**Authors:** Giuseppe Marano, Mariateresa Acanfora, Luca Conci, Gianandrea Traversi, Daniele Napolitano, Osvaldo Mazza, Marianna Mazza

**Affiliations:** 1 Department of Neuroscience, Head-Neck and Chest, Section of Psychiatry, Fondazione Policlinico Universitario Agostino Gemelli IRCCS, Largo Agostino Gemelli 8, 00168 Rome, Italy; mariatacanfora@gmail.com (M.A.);; 2 Department of Neuroscience, Section of Psychiatry, Università Cattolica del Sacro Cuore, Largo Agostino Gemelli 8, 00168 Rome, Italy; 3 Unit of Medical Genetics, Department of Laboratory Medicine, Ospedale Isola Tiberina-Gemelli Isola, 00186 Rome, Italy; gianandrea.traversi@gmail.com; 4 Scientific Direction, Fondazione Policlinico Universitario Agostino Gemelli IRCCS, 00168 Rome, Italy; daniele.napolitano@policlinicogemelli.it; 5 Department of Translational Medicine and Surgery, Università Cattolica del Sacro Cuore, 00168 Rome, Italy; 6 Spine Surgery Department, Bambino Gesù Children’s Hospital IRCCS, 00168 Rome, Italy; osvaldo.mazza1973@hotmail.it

**Keywords:** trauma, hyperarousal, affective disorders, insomnia, nightmares, circadian rhythms, emotional regulation, major depressive disorder, bipolar disorder, PTSD

## Abstract

Sleep disturbances are common across affective disorders and may coexist with traumatic stress and persistent hyperarousal. This narrative review examines how trauma-related hyperarousal and sleep dysfunction are associated with major depressive disorder (MDD) and bipolar disorder (BD), while distinguishing observed associations from proposed mechanisms. A targeted search of PubMed, Scopus, and Google Scholar identified clinical, neurobiological, and translational literature on trauma, hyperarousal, insomnia, nightmares, circadian disruption, MDD, and BD. The evidence supports a multidimensional view of hyperarousal involving partly overlapping autonomic, cognitive–attentional, affective, and behavioral domains; however, it does not establish hyperarousal as a unitary mechanism or justify equating trauma-related vigilance with manic activation. Sleep disturbance may function as a symptom, vulnerability factor, or prodromal change, but no single sleep measure is currently a validated individual-level prognostic marker. Clinically, trauma history, sleep phenotype, affective state, comorbidity, medication effects, and current safety should be assessed jointly. Cognitive behavioral therapy for insomnia and established trauma-focused treatments have the strongest clinical basis within their indicated populations, whereas imagery rehearsal therapy, prazosin, chronobiological strategies, and candidate biomarkers require diagnosis-specific and evidence-graded interpretation. Longitudinal studies integrating objective sleep measures and explicit temporal modeling are needed before proposed pathways or precision approaches can be considered clinically validated.

## 1. Introduction

Mood disorders (MDs) and post-traumatic stress disorder (PTSD) are recurrent psychiatric conditions with substantial individual and public-health consequences. Sleep and circadian disturbances lie at an important clinical intersection: they may occur during an acute affective episode, persist as residual symptoms, precede relapse, or accompany trauma-related threat sensitivity (Geoffroy & Gottlieb, 2020; Geoffroy et al., 2017, 2021; Lewis et al., 2017; Asarnow & Manber, 2019; Macchio et al., 2020; Beaudoin et al., 2023). Prospective studies also suggest that pre-existing sleep problems are associated with poorer outcomes after trauma exposure, although shared vulnerability, prior psychopathology, and unmeasured confounding remain plausible explanations (Neylan et al., 2021; Pruiksma et al., 2016). Hyperarousal is therefore a potentially useful, but conceptually contested, framework for examining the interaction among trauma-related symptoms, sleep disturbance, and affective dysregulation. The present review asks: how are trauma exposure and multidimensional hyperarousal associated with sleep dysfunction in MDD and BD, what mechanisms have been proposed, and which assessment and treatment implications are supported by current evidence? The focus on affective disorders is justified because sleep and circadian changes are integral to episode expression and recurrence in MDD and BD and because trauma-related arousal may be confused clinically with depressive agitation, mixed features, or manic activation. The review accordingly evaluates evidence specific to these diagnoses rather than generalizing from all trauma-exposed populations.

## 2. Materials and Methods

This narrative review was supported by a targeted literature search of PubMed, Scopus, and Google Scholar, completed on 31 August 2026. The principal Boolean combination was: (trauma* OR “post-traumatic stress disorder” OR PTSD OR “childhood adversity”) AND (hyperarousal OR hypervigilance OR “autonomic arousal”) AND (sleep OR insomnia OR nightmare* OR circadian) AND (“major depressive disorder” OR depression OR “bipolar disorder” OR “affective disorder”). The core concepts were retained across databases, while field tags, quotation marks, truncation, and syntax were adapted to the functions available in PubMed and Scopus. Google Scholar was used as a supplementary source to identify additional relevant and citing papers through combinations of the same core terms and relevance-ranked screening. Additional searches paired each diagnostic term with specific sleep phenotypes, neurobiological systems, assessment measures, and interventions. The primary publication window was January 2016–August 2026; earlier seminal studies were retained when necessary to define constructs or contextualize treatment evidence. Eligible English-language sources included systematic reviews, meta-analyses, randomized or controlled trials, longitudinal and cross-sectional observational studies, consensus statements, and clinically relevant mechanistic studies. Conference abstracts, non-peer-reviewed opinion pieces, and sources without direct relevance to trauma, hyperarousal, sleep, or affective disorders were excluded. Two authors (G.M. and M.M.) reviewed titles/abstracts and then potentially relevant full texts; disagreements were resolved by discussion. Eighty-one publications were included in the original synthesis, with one additional pivotal trial added during revision. Because the search was iterative and designed for a narrative rather than systematic review, database yields, duplicate-removal counts, the exact database-specific strings, and the number or cutoff of Google Scholar results screened were not prospectively archived and cannot be reconstructed reliably. No formal risk-of-bias tool or PRISMA flow diagram was used. These features limit reproducibility and permit selection bias. Evidence was synthesized thematically, with explicit distinction among cross-sectional associations, longitudinal findings, experimental evidence, and conceptual hypotheses.

## 3. Conceptualizing Hyperarousal: From Adaptive Vigilance to Persistent Dysregulation

Hyperarousal is not necessarily a unitary construct. It can be described as a continuum from adaptive, time-limited vigilance to persistent dysregulation across partly separable domains: autonomic activation, cognitive–attentional threat monitoring, affective reactivity, and behavior that prevents disengagement from perceived threat (American Psychiatric Association, 2022; Bresser et al., 2026). PTSD nosology illustrates the controversy: the earlier three-cluster model grouped avoidance and numbing, whereas the DSM-5 four-cluster model separates negative alterations in cognition and mood from avoidance and retains a heterogeneous arousal/reactivity cluster (American Psychiatric Association, 2022). Factor-analytic alternatives further suggest that irritability, startle, sleep disturbance, and vigilance need not reflect one latent process. Neuroendocrine findings are similarly nonuniform. PTSD has often been associated with relatively low basal cortisol or enhanced glucocorticoid feedback despite marked clinical arousal, whereas melancholic depression may show hypercortisolemia; chronicity, sampling time, sex, medication, and comorbidity may partly explain this apparent HPA-axis paradox (Palagini et al., 2022; Kizilhan et al., 2026). For this review, hyperarousal is operationalized as persistent or cue-reactive elevation in at least two of three domains, autonomic/somatic activation, cognitive–attentional threat monitoring or inability to disengage, and affective/behavioral reactivity, documented by clinical interview or a validated symptom measure and accompanied by sleep or functional impairment. Rumination, insomnia, or reduced sleep duration alone is insufficient. Trauma-related hyperarousal remains phenomenologically distinct from manic/hypomanic activation, which is characterized by an episodic mood change with increased energy or goal-directed activity and a reduced need for sleep rather than fear-based inability to sleep.

## 4. Trauma-Related Alterations in Sleep

Traumatic exposure is associated with changes in sleep initiation, continuity, timing, dream content, subjective sleep quality, and, less consistently, sleep architecture. The most clinically established patterns are insomnia symptoms and trauma-related nightmares; circadian instability and subjective–objective sleep discrepancy are important but less specifically linked to trauma, while napping is best regarded as a contextual or compensatory behavior rather than a core trauma phenotype (Zhang et al., 2022; Reffi et al., 2023; Baglioni et al., 2016; Farina et al., 2003; Mazza et al., 2020; Paquet et al., 2024; Rosser et al., 2025). Sleep problems observed before a traumatic event may indicate vulnerability, but they may also reflect prior adversity, pre-existing anxiety or mood disorder, socioeconomic stress, medical illness, or measurement overlap. Accordingly, prospective association does not by itself demonstrate that sleep disturbance causes subsequent psychopathology. Objective and subjective measures should be interpreted together because perceived threat and sleep-state misperception may amplify reported disturbance even when polysomnographic abnormalities are modest.

## 5. Neurobiological Pathways Linking Trauma, Hyperarousal, and Sleep

The relationship between trauma, hyperarousal, and sleep dysfunction is mediated by an interconnected brain–body system rather than by a single neurobiological pathway. Acute threat activates sympathetic, endocrine, attentional, and defensive responses that support survival; persistent symptoms may emerge when physiological recovery is incomplete and threat-detection systems remain readily reactivated. Dysregulation of the hypothalamic–pituitary–adrenal axis may alter diurnal cortisol secretion, the cortisol-awakening response, glucocorticoid sensitivity, and the temporal coordination between endocrine activity and the sleep–wake cycle. However, the direction and magnitude of these alterations vary according to trauma type, developmental timing, chronicity, affective state, medication exposure, and comorbid medical conditions (Palagini et al., 2022; Kizilhan et al., 2026). Persistent sympathetic activation and reduced parasympathetic flexibility may further limit nocturnal recovery, although indices such as heart-rate variability are influenced by age, physical fitness, respiration, medication, and medical illness and cannot currently be considered diagnostic biomarkers in isolation (Kizilhan et al., 2026; Dikeos et al., 2023). At the neural level, heightened amygdala responsivity together with reduced recruitment of medial prefrontal and anterior cingulate regulatory regions provides a plausible model for persistent threat detection and impaired fear extinction, while hippocampal dysfunction may weaken contextual discrimination between past threat and present safety. Sleep loss can further reduce top-down cognitive control and increase limbic reactivity, establishing a reciprocal loop in which hyperarousal disrupts sleep and insufficient sleep lowers the threshold for emotional responses (Baglioni et al., 2016; Palagini et al., 2022). Altered coordination among salience, default-mode, and executive-control networks may contribute to excessive threat attribution, intrusive recollection, rumination, and difficulty disengaging from internal or external cues at bedtime; nevertheless, these circuit-level findings remain group-level associations and do not provide validated diagnostic cutoffs for individual patients (Dikeos et al., 2023). Noradrenergic overactivity, particularly within the locus coeruleus system, may increase vigilance, impair NREM continuity, facilitate rapid awakenings, and intensify autonomic activation during trauma-related nightmares, whereas serotonergic, dopaminergic, and GABAergic alterations may affect mood, inhibitory control, reward processing, and sleep timing. Neuroinflammatory pathways provide an additional link: chronic stress and sleep loss can promote low-grade inflammatory signaling, while inflammatory mediators may affect monoaminergic transmission, neuroplasticity, sleepiness, and sleep continuity (Li et al., 2026; Pan, 2026). The gut–brain axis may participate through immune regulation, intestinal-barrier integrity, tryptophan metabolism, and microbial metabolites; however, the therapeutic use of prebiotics remains supported more strongly by mechanistic plausibility than by robust psychiatric trials (Marano et al., 2026a). Trauma may also destabilize the interaction between circadian phase and homeostatic sleep pressure through nocturnal vigilance, irregular light exposure, reduced activity, altered meal timing, and affective episodes. Chrono-metabolic models extend this framework to reciprocal interactions among circadian rhythms, microbial oscillations, and host metabolism, but their clinical utility remains to be established prospectively (Marano et al., 2026a). Finally, REM sleep is relevant to emotional-memory integration and fear extinction, and persistent noradrenergic activation during REM may contribute to vivid nightmares and abrupt awakenings; nonetheless, REM and NREM findings are heterogeneous and must be interpreted in relation to diagnosis, medication, comorbidity, and laboratory conditions (Baglioni et al., 2016; Farina et al., 2003; Palagini et al., 2022). These interacting mechanisms support multimodal assessment while underscoring that no individual neuroendocrine, inflammatory, autonomic, electrophysiological, or neuroimaging measure is currently sufficiently validated for routine diagnosis or treatment selection (Dikeos et al., 2023).

## 6. Cognitive and Behavioral Mechanisms

Cognitive and behavioral processes may maintain both insomnia and trauma-related symptoms, but trauma introduces features not captured fully by standard insomnia models. In addition to conditioned arousal, sleep effort, and maladaptive beliefs, the bed or darkness may become a cue for traumatic memories; relinquishing vigilance may be experienced as unsafe; nightmares may motivate deliberate sleep avoidance; and nocturnal bodily sensations or environmental sounds may be interpreted as signals of renewed threat (Reffi et al., 2023). These processes can reinforce checking, safety behaviors, irregular schedules, and substance use. Thus, the relevant formulation is not simply excessive worry about sleep but learned threat prediction attached to sleep and the sleeping environment. This distinction supports assessment of trauma cues, current safety, nightmare content, and dissociative symptoms alongside conventional insomnia-maintenance factors.

## 7. Bidirectional Relationships Between Sleep Disturbance and Affective Dysregulation

Sleep and affective functioning are linked through reciprocal processes involving mood, emotional reactivity, and emotion regulation. Hyperarousal can interfere with sleep initiation and continuity, while insufficient or fragmented sleep can increase negative affect, weaken cognitive control, and reduce the capacity to regulate emotional responses. These interactions are clinically relevant across PTSD, depressive disorders, and BD, although their expression varies according to developmental stage, sex, baseline vulnerability, comorbidity, and the cause and duration of sleep disruption (Geoffroy & Gottlieb, 2020; Bresser et al., 2026; Mazza et al., 2022 Marano & Mazza, 2026). Rather than representing a simple unidirectional sequence, trauma-related hyperarousal, sleep disturbance, and affective dysregulation can progressively reinforce one another.

### 7.1. Effects of Hyperarousal on Sleep

Persistent cognitive–attentional and autonomic arousal is associated with delayed sleep onset, fragmented sleep, and difficulty returning to sleep. Anticipatory anxiety, threat monitoring, intrusive recollection, rumination, and heightened attention to bodily or environmental cues may impede disengagement from wakefulness. These associations are consistent with a bidirectional maintenance model, but most clinical evidence does not establish a single causal pathway. To avoid conceptual duplication, the differential diagnosis between trauma-related hyperarousal and manic/hypomanic activation is addressed once, in Section 10.3.

### 7.2. Effects of Sleep Disturbance on Affective Regulation: A Bidirectional Cycle

Insufficient, fragmented, or irregular sleep is associated with increased negative affect, irritability, impulsivity, and stress sensitivity. Experimental sleep restriction supports short-term effects on emotional reactivity, whereas the direction and magnitude of longer-term relationships in clinical populations are influenced by illness severity, medication, substance use, and social context. Sleep disturbance may therefore contribute to affective dysregulation, result from it, or participate in a reciprocal cycle; the available evidence should not be interpreted as proving that sleep disruption alone causes an affective episode (Figure 1).

## 8. Trauma, Hyperarousal, and Sleep in Depressive Disorders

### 8.1. Trauma Exposure and Sleep Disturbance in MDD

Sleep problems are listed among the main criteria and manifestations of MDD and can substantially impair functioning (Stanners et al., 2014). Insomnia is also frequently reported by trauma-exposed patients (Moore et al., 2023). The prevalence of insomnia among people with depression has been estimated at approximately 90.9% in the cited sample (Seow et al., 2016). Diagnoses of MDD and PTSD are also reported in military medical settings (Moore et al., 2023). Some service personnel or veterans may initially present with physical or physiological complaints, including insomnia, rather than spontaneously disclosing emotional distress or traumatic memories (Moore et al., 2023). Insomnia should therefore prompt assessment for possible PTSD, MDD, and other psychiatric or medical conditions, but it is a nonspecific symptom and should not be regarded as an individual-level diagnostic or prognostic marker. Wang et al. reported that, although PTSD is a common outcome following trauma, MDD may also develop without concomitant PTSD, particularly after repeated exposure to stressful events (Z. Wang et al., 2010). During the COVID-19 pandemic, trauma symptoms, MDD, and sleep disorders frequently co-occurred in population studies, including work from Saudi Arabia and Italy (Casagrande et al., 2020). Large-scale disruptions such as the pandemic illustrate how threat, social isolation, altered routines, reduced activity, and sleep disturbance can coexist with depressive and anxiety symptoms. These findings are contextually informative but do not establish that all pandemic-related distress was traumatic or that sleep mediated every mental-health outcome (Caroppo et al., 2021).

### 8.2. Insomnia and Hyperarousal in MDD

The bidirectional link between stress and insomnia is well documented: both disruption of sleep homeostasis increases HPA axis activity and glucocorticoids directly alter sleep (Nollet et al., 2020). This creates a vicious circle that further exacerbates depressive symptoms (X. Wang et al., 2024). The transition from childhood to adolescence alone involves a physiological reduction in sleep duration, characterized by delayed sleep onset and a shortening of REM sleep latency (Gaudreau et al., 2001). Furthermore, compared with healthy individuals, patients with MDD exhibit delayed or irregular sleep–wake cycles, longer sleep onset latency and more night-time awakenings (Tesler et al., 2015). The presence of MDD has been associated with increased REM sleep pressure and marked inhibition of sleep depth, leading to early awakenings (Baglioni et al., 2016). Insomnia therefore indicates a transdiagnostic imbalance in the arousal system (Baglioni et al., 2016). Polysomnographic macrostructure does not fully capture arousal instability. Cyclic alternating pattern analyses have identified NREM microstructural alterations in depressed patients, supporting the view that sleep instability may persist beneath conventional stage summaries (Farina et al., 2003).

### 8.3. Nightmares, Rumination, and Depressive Symptom Severity

Nightmares are vivid, intensely unpleasant or frightening dreams characterized by negative emotions such as anxiety, fear, and a sense of threat; they may be followed by abrupt awakening and a startle response (American Psychiatric Association, 2022). Women report nightmares more frequently than men across age groups (Sandman et al., 2013). Nightmares are associated with sleep disruption and daytime emotional difficulties. In MDD, greater nightmare frequency or distress has been linked to negative affect and poorer clinical outcomes, although directionality and underlying mechanisms remain uncertain (X. Wang et al., 2018). The emotional cascade model describes a self-reinforcing association between negative emotions, including anxiety, depression, and hyperarousal, and rumination about those emotions (Selby et al., 2009). Higher rumination is associated with greater cognitive arousal and nightmare distress (Selby et al., 2009). Wang et al. reported that depression-related rumination mediated the association between neuroticism and dream anxiety in their sample; this statistical mediation does not by itself establish a causal sequence (X. Wang et al., 2024). Brooding is also associated with suicidal ideation and suicide attempts (Miranda & Nolen-Hoeksema, 2007; Rogers et al., 2017). Rogers et al. found correlations among rumination, manifestations of hyperarousal, and suicidal ideation and proposed that brooding may contribute to suicidal thoughts or behavior through overarousal; this remains a proposed pathway rather than an established mechanism (Rogers et al., 2017).

### 8.4. Sleep as a Predictor of Persistence, Recurrence, and Treatment Response

Sleep disturbance in MDD should be described according to its temporal and evidentiary role. It may be a concurrent symptom, a residual symptom associated with recurrence risk, or a nonspecific vulnerability factor. A change that precedes an episode in an individual may be considered a possible prodromal symptom, whereas a prognostic marker requires prospectively validated thresholds, sensitivity, specificity, and incremental predictive value. These requirements have not been met for any single sleep parameter in routine MDD care. Persistent insomnia is nevertheless clinically relevant because group-level studies associate it with symptom persistence, impaired functioning, and suicidality (Geoffroy & Gottlieb, 2020; Geoffroy et al., 2021; Seow et al., 2016; Hertenstein et al., 2022); such associations support monitoring and treatment but not deterministic individual prediction.

## 9. Trauma, Hyperarousal, and Sleep in BD

### 9.1. Childhood and Adult Trauma in BD

Childhood trauma (CT), which includes abuse, neglect, and family dysfunction before the age of 18, is strongly associated with bipolar disorder (BD) and with greater symptom severity, rapid cycling, suicidal behavior, and comorbidity (Thurston et al., 2023). The systematic review by Lim et al. (2025) examined associations between types of CT and features of BD: sexual abuse (SA) was associated with greater severity, more annual manic/hypomanic and depressive episodes, and rapid cycling, including ultra- and ultra-ultra-rapid cycling; physical abuse (PA) correlated with hypomanic symptoms and rapid/ultra-rapid cycles; suicidal ideation was more frequent with SA and PA and highest when both were reported; greater trauma burden was associated with relapse after mild stress; any form of childhood trauma was associated with more involuntary hospitalizations; and histories of SA, PA, or emotional abuse (EA) were associated with greater functional impairment. Neurobiological studies suggest that PA and SA may be associated with marked HPA-axis dysregulation and altered stress responsivity, whereas EA has been linked to differences in cognitive processes, identity development, attachment, and resilience; these findings do not establish trauma-type-specific causal pathways (Lim et al., 2025). Compared with people with MDD, those with BD report a higher frequency of stressful life events, although these events do not always meet full PTSD criteria. In addition to higher levels of CT, patients with BD have higher reported rates of physical or sexual assault, disasters, or crime exposure in adulthood (Rowe et al., 2024; McCraw & Parker, 2017; Theodoratou & Puri, 2025). Adult trauma has received less study and is often examined in the context of comorbid PTSD, which is common in BD (Cerimele et al., 2017). Wrobel et al. found that CT alone, or CT combined with adult trauma, but not adult trauma alone, was associated with greater depression severity at 2- and 6-year follow-up (Wrobel et al., 2023).

### 9.2. Sleep and Circadian Instability Across Mood States

The biological clock contributes to the regulation of mood and motivation, and sleep and circadian disturbances are common in MDD and BD; acute or repeated stress is also associated with circadian disruption (Marano et al., 2026b). In MDD, reported abnormalities include insomnia, altered circadian phase or amplitude, diurnal symptom variation, and changes in appetite, blood pressure, body temperature, and hormone secretion. In BD, circadian instability may include irregular sleep timing, evening preference, and altered melatonin secretion, with separate studies reporting associations with anxiety, functioning, mood variability, inflammatory measures, or BMI (Marano et al., 2026c). The nonstandard expression “serotoninic chronotype” has therefore been removed, and these heterogeneous findings should not be interpreted as one established clinical phenotype.

### 9.3. Hyperarousal During Depression, Hypomania, Mania, and Mixed States

The cross-sectional study by Sato et al. assessed intrusion, avoidance, and hyperarousal symptoms reported following stressful life events. Patients with BD in acute manic/hypomanic or depressive phases had higher hyperarousal scores than euthymic patients, both for stressful events generally and for events perceived to have preceded illness onset; hyperarousal severity was also positively associated with depressive-symptom severity (Sato et al., 2018). Because the design was cross-sectional, these findings do not establish temporal or causal direction. Depressive severity may be associated with greater recall of and rumination on negative memories, but proposed prefrontal-amygdala mechanisms remain inferential (Sato et al., 2018). Bresser et al. described irritable and anxious dimensions of hyperarousal in MDD: the former includes reactive tension, irritability, agitation, and hostility, whereas the latter includes anticipatory anxiety, apprehension, persistent worry, and perceived threat (Bresser et al., 2026). Mixed states have been associated with altered monoaminergic, HPA-axis, inflammatory, and circadian measures compared with unmixed presentations, although findings are heterogeneous and do not establish a single biological mechanism (Muneer, 2017). Table 1 summarizes reported manifestations of hyperarousal across mood states and the proposed clinical or neurobiological correlates.

### 9.4. Sleep Loss and Affective Switching

Sleep loss has been reported as a possible precipitant of mood episodes, particularly mania, but susceptibility varies and the evidence is not deterministic (Jackson et al., 2003; Jermann et al., 2022). In the Bipolar Disorder Research Network sample, Lewis et al. found that patients with BD-I were more likely than those with BD-II to report mania/hypomania following sleep loss. Women more often reported mania/hypomania or depression after insufficient sleep than men. These retrospective, self-reported associations support individualized monitoring but do not show that sleep loss invariably causes relapse (Lewis et al., 2017).

### 9.5. Trauma-Related Nightmares and Affective Instability

Nightmares may function as recurrent nocturnal stressors and are associated with next-day emotional difficulty, autonomic activation, abrupt awakening, fragmented sleep, insomnia, and fear of sleep; proposed sensory, limbic, and autonomic mechanisms remain inferential rather than established sequential effects (Paquet et al., 2024). Trauma-related nightmares often emerge within the first months after trauma and are common in PTSD, although prevalence varies by population, trauma type, and assessment method (Zhang et al., 2022). They are also associated at the group level with poorer sleep quality, depressive symptoms, and suicidal risk (Zhang et al., 2022).

### 9.6. Sleep Disturbance as a Predictor of Relapse and Functional Outcomes

In BD, reduced sleep duration or circadian disruption may precede some manic, depressive, or mixed episodes and can therefore serve as an individualized warning sign when it represents a reproducible change from baseline (Lewis et al., 2017; Jackson et al., 2003). It should not be labeled a validated relapse marker in the absence of established thresholds and adequate sensitivity, specificity, and independent prognostic performance. Reduced need for sleep with preserved energy is more suggestive of manic activation, whereas distressing inability to sleep with vigilance or fear is more consistent with insomnia or trauma-related arousal. Prospective monitoring may aid collaborative relapse planning, but sleep data must be interpreted with mood symptoms, medication adherence, substance use, and contextual stressors (Figure 2).

## 10. Post-Traumatic Symptoms and Affective Disorders: Diagnostic Overlap and Comorbidity

### 10.1. PTSD Symptoms Within Depressive and Bipolar Presentations

PTSD shares symptoms with mood disorders, which can complicate differential diagnosis and clinical management. Overlapping features may include anhedonia, guilt, social withdrawal, hopelessness, insomnia, early awakening, fatigue, and cognitive complaints in MDD, as well as hyperarousal, irritability, psychomotor agitation, emotional reactivity, anger, impulsive behavior, insomnia, and nightmares in hypomanic or mixed presentations (American Psychiatric Association, 2022). PTSD may be under-recognized in services treating severe psychiatric disorders, including schizophrenia, BD, and MDD, particularly when another diagnosis dominates the clinical presentation (Lu et al., 2024). Comorbid PTSD and BD are associated with greater symptom severity, substance misuse, psychotic features, and suicidal risk, while comorbid MDD and PTSD are associated with greater functional impairment and poorer prognosis; these associations support integrated assessment rather than a presumed causal relationship between the diagnoses (Lu et al., 2024).

### 10.2. Shared and Relatively Characteristic Sleep Abnormalities

Several sleep disturbances are transdiagnostic, including difficulty initiating or maintaining sleep, early-morning awakening, and subjective hyperarousal (Palagini et al., 2022). DSM-5 accordingly allows insomnia disorder to be diagnosed independently or comorbidly with another mental disorder (Hertenstein et al., 2022). Some features are relatively characteristic of particular disorders, but their evidentiary status differs. Reduced need for sleep with preserved energy is a diagnostic symptom of mania/hypomania. By contrast, shortened REM latency or altered REM density in MDD and motor or sympathetic activation during REM in PTSD are heterogeneous group-level polysomnographic findings influenced by medication, comorbidity, sampling, and laboratory conditions (Lewis et al., 2017; X. Wang et al., 2024; Lu et al., 2024). They do not have sufficient specificity for individual differential diagnosis. Clinical interpretation should therefore integrate symptom pattern, longitudinal course, mood state, trauma history, medication, and comorbidity rather than infer diagnosis or relapse risk from a single physiological sleep feature. A two-sample Mendelian-randomization study reported findings compatible with bidirectional effects between insomnia and MDD; this method can strengthen causal inference under its instrumental-variable assumptions but does not establish a deterministic relationship in individual patients (Huang et al., 2021).

### 10.3. Differentiating Trauma-Related Hyperarousal from Manic Activation

Trauma-related hyperarousal and manic/hypomanic activation may both involve insomnia, irritability, motor restlessness, and autonomic activation, but their organizing phenomenology differs. Trauma-related arousal is typically cue-linked or fear-based and includes vigilance, exaggerated startle, intrusive recollection, and distress about inability to sleep. Manic/hypomanic activation requires an episodic change in mood and energy or goal-directed activity and commonly features a reduced need for sleep without proportional fatigue, together with pressured speech, grandiosity, or risk taking. Mixed states can blur this distinction, and PTSD and BD may coexist (Cerimele et al., 2017; Muneer, 2017; Russell et al., 2023). Diagnosis should therefore integrate longitudinal course, relation to trauma cues, sleep need versus sleep ability, collateral history, substances, and medication effects rather than relying on arousal symptoms alone.

### 10.4. Diagnostic Masking and Misattribution: Clinical Implications of Psychiatric Comorbidity

Dunphy et al. carried out a retrospective analysis of data on 19.1 million insured individuals in the US: those who had been assaulted were 98.4 per cent more likely than the control group to develop a psychiatric disorder; the highest relative increase was recorded for PTSD, with a 53.0 per cent increase for unintentional injuries and a 354.9 per cent increase for assaults (Dunphy et al., 2026). The presence of multiple disorders can mask or confound symptoms: in particular, it appears that PTSD is highly likely to be completely overlooked in white males and in patients with psychotic or bipolar disorders (Dunphy et al., 2026). The presence of BD or PTSD constitutes a risk factor for the onset of the other, and the co-occurrence of the two disorders results in a significantly higher symptom burden and poorer QoL compared with the presence of a single condition; furthermore, the co-occurrence of two psychiatric disorders makes treatment more difficult (Cerimele et al., 2017). Patients with comorbid insomnia, compared with those suffering from a mental disorder without insomnia, are characterized by a more unfavorable course of the disorder and lower overall treatment efficacy (Seow et al., 2018). The same principle applies beyond psychiatric comorbidity. In breast cancer, insomnia can cluster with fatigue, distress, pain, treatment effects, and circadian disruption; treating it as a minor accompaniment risks overlooking a clinically meaningful and potentially modifiable burden (Marano et al., 2026b). This oncology literature is not direct evidence for trauma-related mechanisms, but it illustrates why insomnia should be assessed as an interacting symptom domain rather than automatically attributed to the primary diagnosis.

## 11. Clinical Phenotypes and Sources of Heterogeneity

Trauma-related sleep disturbance is heterogeneous. The most directly relevant patterns combine sleep-onset or sleep-maintenance insomnia, trauma-related nightmares, vigilance, and affective symptoms; circadian irregularity and subjective–objective discrepancy may modify presentation but are less trauma-specific. Heterogeneity also reflects diagnosis, current mood state, sex, developmental stage, medical comorbidity, medication, substance use, and social context. Evidence from oncology, pandemic conditions, or digital-media exposure can illustrate general contextual modifiers of sleep, but it does not constitute direct evidence for trauma-specific causation and is therefore not used here to support the central model. Table 2 provides a conceptual organization of assessment patterns for hypothesis generation. Its categories are neither validated diagnostic subtypes nor a classification system, and the listed interventions are examples supported to varying degrees in broader indicated populations rather than validated phenotype-specific treatment algorithms.

## 12. Clinical Assessment

A comprehensive trauma-informed psychiatric assessment should integrate structured diagnostic interviews, validated symptom scales and, where appropriate, objective measures. Cultural and linguistic background, previous interpersonal violence, current safety, dissociative symptoms and help-seeking behaviors should also be considered, as they may influence symptom expression, the reliability of the information collected and the development of a safe therapeutic relationship. Sleep is a particularly important domain. Wearable actigraphy enables objective, longitudinal monitoring of sleep–wake patterns in everyday life and may identify associations between poor sleep, fatigue and biomarkers related to inflammation and neuroplasticity that are relevant to recovery (Smith et al., 2024). Trauma-related sleep symptoms commonly include nightmares, nocturnal autonomic arousal and sleep-related hypervigilance. The 11-item Seattle Trauma and Autonomic Reactivity Sleep Symptoms Scale (STARSS) was developed to assess these features and was designed to capture these features, although its psychometric performance and incremental validity require replication before it can be considered more specific than generic insomnia measures (Rosser et al., 2025). Established tools such as the Insomnia Severity Index and the Pittsburgh Sleep Quality Index remain useful for assessing sleep initiation, maintenance, perceived sleep quality and daytime impairment. However, their use in culturally and linguistically diverse populations requires careful adaptation, and clinician-led interviews supported by bilingual therapists or trained interpreters may improve assessment accuracy (Kizilhan et al., 2026). Objective measures such as polysomnography and actigraphy provide complementary information beyond self-report. Increased wake after sleep onset, reduced sleep efficiency and lower relative amplitude have been associated with cognitive impairment and subjective sleep complaints, supporting the use of actigraphy for longitudinal monitoring (Milman et al., 2022). Assessment should also consider circadian disruption, hyperarousal and physiological reactivity through validated PTSD measures and indicators such as heart-rate variability and electrodermal activity. Candidate biomarkers, including cyclic alternating pattern indices, rest-activity measures, brain-derived neurotrophic factor concentrations and neuroimaging findings, remain promising but require further validation before routine clinical use (Dikeos et al., 2023). Ecological momentary assessment may further improve evaluation by capturing real-time changes in sleep, mood, dissociation and hyperarousal. Suicide-risk assessment should also be systematically included, particularly when sleep disturbance, depression, impulsivity or hyperarousal are present. CT, depression severity, impulsivity and hostility may contribute to suicidal ideation through distinct pathways, with sleep disturbance acting as a partial mediator (Alter et al., 2021). Overall, objective technologies and biomarkers should complement, rather than replace, a culturally sensitive clinical interview and a strong therapeutic alliance. A 24-h history should explicitly include naps because napping may indicate compensatory sleep, hypersomnolence, avoidance, medication effects, or reduced homeostatic pressure (Mazza et al., 2020).

## 13. Evidence-Based Treatment

Treatment should be matched to diagnosis, symptom target, current mood state, and patient preference, and the strength of evidence should be stated explicitly. Established or guideline-supported care includes cognitive behavioral therapy for insomnia (CBT-I) for chronic insomnia, appropriately adapted when PTSD or BD is present, and established trauma-focused psychotherapies for core PTSD symptoms (Jia et al., 2026). Imagery rehearsal therapy (IRT) may reduce recurrent nightmares, but evidence for a combined CBT-I–IRT protocol in trauma-exposed populations includes a case series, a meta-analysis with heterogeneous studies, and a pilot randomized trial (Bishop et al., 2015; Casement & Swanson, 2012; Prguda et al., 2023); it therefore supports a promising option rather than a universally recommended combination. Interpersonal and social rhythm approaches may be useful for rhythm stabilization in BD, but they are not specific treatments for trauma-related hyperarousal. Pharmacotherapy requires particular caution. Prazosin has shown benefit for nightmares in some studies, yet the large 2018 VA Cooperative trial found no advantage over placebo for distressing dreams or sleep quality in military veterans (Raskind et al., 2018); current evidence is mixed, and response may vary by population. Melatonin may be considered for circadian-timing problems or selected insomnia presentations, but evidence specific to trauma-related sleep disturbance is limited. SSRIs are evidence-based for PTSD and MDD, not as primary hypnotics, and antidepressant monotherapy may precipitate mood destabilization in BD. Lamotrigine is used for bipolar depression and maintenance but is not an established treatment for trauma-related insomnia or hyperarousal. Trazodone is frequently prescribed for insomnia, although trauma-specific efficacy and long-term benefit–risk evidence are limited. Microbiota-directed interventions remain experimental and should not be presented as clinical treatment for this indication. Medication decisions require diagnostic clarification, attention to adverse effects and interactions, and monitoring for affective switching or suicidality (Marano et al., 2023).

## 14. Emerging Biomarkers and Translational Research

Objective measures are being investigated as research-level candidate biomarkers of trauma-related sleep disturbance, including wearable-derived sleep timing and variability, quantitative EEG or event-related potentials, inflammatory measures, autonomic indices, and PSG microstructure (Milman et al., 2022; Straus et al., 2023; Casazza et al., 2025; Lanir-Azaria, 2026; Smagula et al., 2016). None currently has sufficiently replicated thresholds, diagnostic specificity, calibration, or prospective clinical utility to be considered a validated biomarker for routine assessment or treatment selection. Wearables may support longitudinal description of sleep–wake patterns, but device error, algorithm opacity, adherence, and population differences limit interpretation. Candidate signals should therefore be reported as exploratory correlates or predictors until they are prospectively validated against prespecified clinical outcomes and shown to add value beyond standard assessment.

## 15. An Integrated Neurobehavioral Model

The proposed neurobehavioral model is best understood as a heuristic synthesis rather than a causal model. Predisposing vulnerability, trauma exposure, threat appraisal, sleep disruption, affective state, and contextual factors may interact over time, but the direction and strength of these relationships vary across individuals and are incompletely established. Longitudinal and experimental evidence supports some component pathways, whereas many links remain cross-sectional associations or biologically plausible hypotheses. The model is intended to organize assessment and research questions, not to imply a single mechanism shared by PTSD, MDD, and BD.

## 16. Limitations of the Current Evidence

The evidence base has important limitations. Many studies are cross-sectional, use retrospective trauma reports or self-reported sleep, and include heterogeneous diagnostic and treatment contexts. These features limit temporal inference and make residual confounding likely. Definitions and measures of hyperarousal differ substantially, and autonomic activation, rumination, vigilance, insomnia-related arousal, and manic activation are not interchangeable. Intervention evidence is uneven, frequently diagnosis-specific, and often based on small or selected samples. This narrative review also used an iterative search without prospectively recorded screening counts or formal risk-of-bias assessment, increasing the possibility of selection and interpretation bias. Consequently, associations and proposed mechanisms summarized here should not be interpreted as established causal pathways, validated phenotypes, or individual-level prediction tools.

## 17. Future Research Directions

Future research should focus on priorities that follow directly from the current evidence. First, longitudinal studies beginning before or soon after trauma exposure should combine repeated clinical assessment with actigraphy or PSG to clarify temporal relationships among trauma symptoms, hyperarousal domains, sleep phenotypes, and affective episodes. Second, studies should test explicit, reproducible definitions of hyperarousal and determine whether autonomic, cognitive–attentional, affective, and behavioral components show shared or distinct trajectories. Third, samples should be stratified prospectively by MDD versus BD, mood state, sex, and developmental stage, with medication, substance use, and prior adversity measured as potential confounders or moderators. Fourth, proposed biomarkers and digital measures require external validation, prespecified thresholds, calibration, and demonstration of incremental clinical utility. Finally, adequately powered trials should evaluate diagnosis-specific sleep and trauma interventions, report harms and affective switching, and identify replicable treatment-response moderators rather than assuming that one transdiagnostic protocol applies across disorders.

## 18. Conclusions

Sleep disturbance and trauma-related hyperarousal are clinically important, partly overlapping dimensions across PTSD and affective disorders, but the available evidence does not establish a single causal pathway or a unitary transdiagnostic mechanism. Clinically supported practice includes careful assessment of trauma history, current safety, sleep phenotype, affective state, comorbidity, substance use, and medication effects. Sleep changes may be symptoms, vulnerability factors, or individualized warning signs; they should not be treated as validated prognostic markers without prospective evidence. CBT-I and established trauma-focused therapies should be used within their evidence-based indications, while adjunctive pharmacological and nightmare-focused interventions require individualized, diagnosis-specific appraisal of mixed or limited evidence. Wearable, physiological, inflammatory, and neurophysiological measures remain research candidates rather than clinically validated biomarkers. More rigorous longitudinal studies and adequately powered trials are needed before precision-stratified models can be translated into routine care.

## Figures and Tables

**Figure 1:**
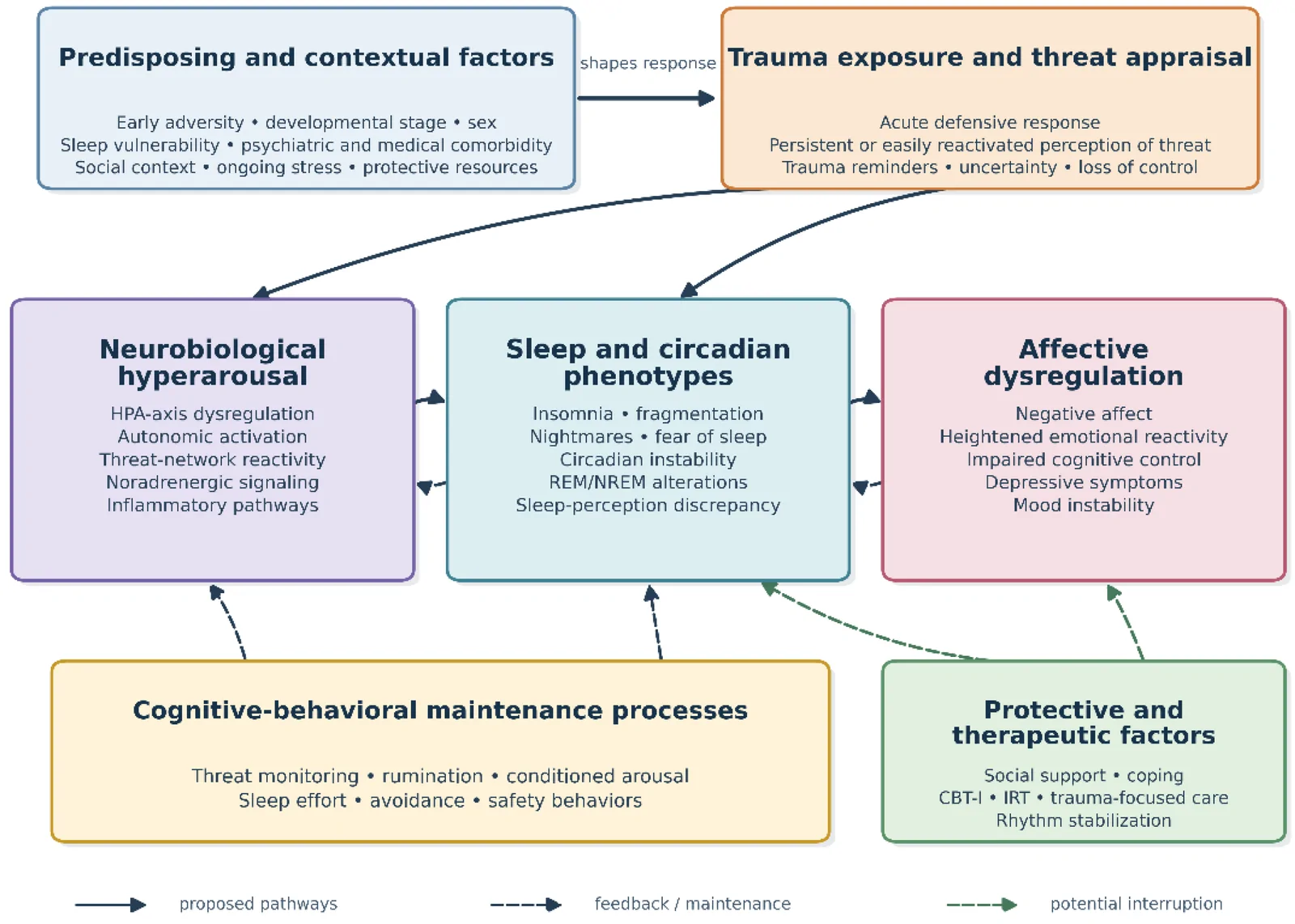
Integrated neurobehavioral model linking trauma, hyperarousal, sleep disturbance, and affective dysregulation. Predisposing and contextual factors may influence the individual response to trauma and the persistence of threat appraisal. Trauma-related activation of neuroendocrine, autonomic, neural, inflammatory, and circadian systems may contribute to insomnia, nightmares, sleep fragmentation, fear of sleep, and irregular sleep timing. Sleep disturbance may, in turn, increase negative affect, emotional reactivity, cognitive-control difficulties, and mood instability, potentially reinforcing hyperarousal and threat sensitivity. Cognitive-behavioral processes, including rumination, conditioned arousal, sleep effort, avoidance, and safety behaviors, may maintain these reciprocal relationships. Protective factors and integrated interventions may interrupt the cycle at multiple levels. This figure is a conceptual representation and does not depict a validated causal model or clinical algorithm.

**Figure 2:**
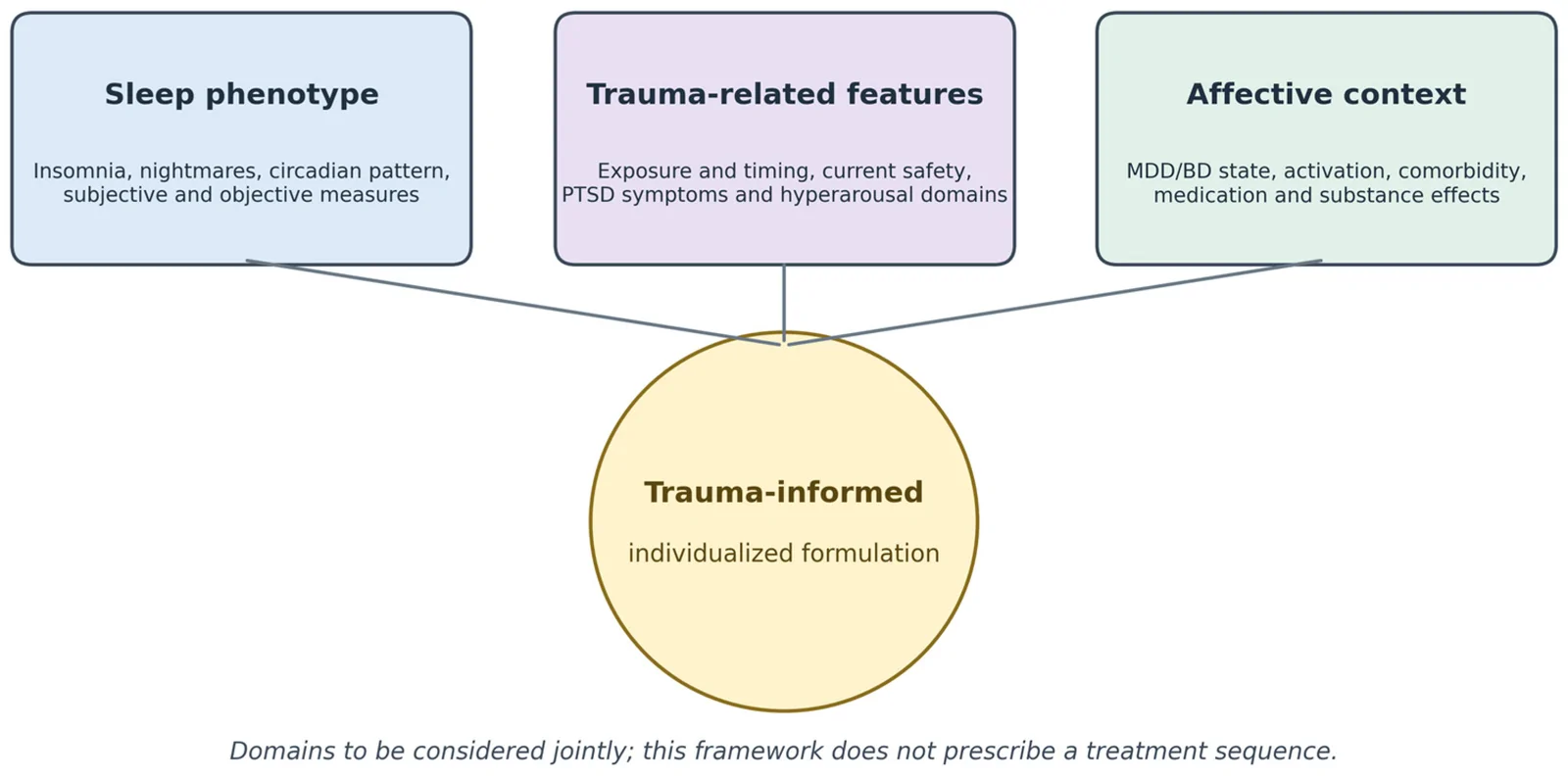
Non-prescriptive domains for a trauma-informed formulation of sleep disturbance in affective disorders. Note. Sleep phenotype, trauma-related features, and affective context should be considered jointly to inform individualized care. The figure is a conceptual framework and does not represent a validated sequence, decision rule, or treatment algorithm.

**Table 1:** Hyperarousal in mood disorders.

Clinical Condition	Manifestations of Hyperarousal	Neurobiological Mechanisms & Clinical Impact
Depression	Irritable: Reactive tension, irritability, agitation, hostility, “fight/angry” response Anxious: Anticipatory anxiety, apprehension, constant worry, feeling of “impending threat”	Maps the different clinical expressions of internal tension: externalized (irritability/anger) vs. internalized (anxiety/apprehension).
Bipolar Disorder	Significantly higher levels in manic/hypomanic and depressive phases compared to the euthymic state	Strong positive association with the severity of depressive symptoms. Rumination on negative memories maintains elevated hyperarousal.
Mixed States	Hyperarousal serves as a hallmark feature.	Elevated levels of central and peripheral monoamines. Greater HPA axis alterations. Increased inflammatory state. Pronounced dysfunction of circadian rhythms.

Abbreviations. HPA: hypothalamic–pituitary–adrenal.

**Table 2:** Conceptual organization of trauma-related sleep presentations for assessment and hypothesis generation; categories and treatment options are not validated phenotype-specific algorithms.

Clinical Phenotype	Core Clinical Features	Predominant Maintaining Processes	Suggested Assessment	Potential Evidence-Informed Options (Not Phenotype-Specific Recommendations)
Sleep-onset insomnia with cognitive hyperarousal	Prolonged sleep-onset latency; racing thoughts; rumination; anticipatory anxiety	Threat monitoring; repetitive negative thinking; sleep effort; catastrophic beliefs about sleep	Clinical sleep interview; ISI; sleep diary; assessment of rumination and pre-sleep arousal	CBT-I; cognitive restructuring; stimulus control; relaxation or mindfulness-based strategies
Sleep-maintenance insomnia with autonomic hyperarousal	Recurrent awakenings; palpitations; sweating; heightened sensitivity to environmental stimuli	Sympathetic activation; nocturnal vigilance; conditioned arousal; pain or medical comorbidity	Sleep diary; PSQI; autonomic-symptom assessment; actigraphy; PSG when clinically indicated	CBT-I; reduction in nocturnal safety behaviors; treatment of pain and medical comorbidities; targeted pharmacotherapy when appropriate
Trauma-related nightmare phenotype	Recurrent distressing dreams; abrupt awakenings; autonomic activation; difficulty returning to sleep	Impaired emotional-memory processing; fear conditioning; anticipatory anxiety; REM instability	Nightmare frequency and distress; dream diary; PTSD assessment; STARSS	IRT; trauma-focused psychotherapy; CBT-I; prazosin when clinically appropriate
Fear-of-sleep and nocturnal-vigilance phenotype	Bedtime avoidance; fear of losing control; sleeping with lights on; repeated checking	Conditioned threat responses; safety behaviors; experiential avoidance; fear of vulnerability	Fear-of-sleep assessment; behavioral analysis of bedtime routines; PTSD symptom measures	Graded modification of safety behaviors; trauma-informed exposure; CBT-I; psychoeducation
Circadian-instability phenotype	Delayed or irregular sleep timing; marked night-to-night variability; daytime sleepiness; irregular activity and meal timing	Circadian misalignment; inconsistent zeitgebers; nighttime light exposure; reduced daytime activity	Seven- to fourteen-day sleep diary; actigraphy; chronotype assessment; review of light exposure and meal timing	Regularization of sleep–wake and social rhythms; morning light exposure; activity scheduling; interpersonal and social rhythm therapy
Subjective–objective sleep-discrepancy phenotype	Severe perceived insomnia despite relatively preserved objective sleep duration	Heightened sleep monitoring; altered sleep perception; fragmented light sleep; memory bias	Sleep diary combined with actigraphy or PSG; assessment of sleep-related beliefs	Sleep education; reduction in sleep monitoring; CBT-I; feedback based on objective measures when therapeutically appropriate
Short-sleep-duration insomnia phenotype	Persistent insomnia with objectively reduced sleep duration and prominent daytime impairment	Physiological hyperarousal; increased homeostatic and cardiometabolic burden	Sleep diary; actigraphy or PSG; cardiometabolic assessment	CBT-I; management of medical risk factors; careful longitudinal monitoring
Mood-state-related sleep disturbance	Insomnia and early awakening in depression; reduced need for sleep in mania; irregular sleep in mixed states	Affective activation; circadian instability; medication effects; interaction between sleep loss and mood regulation	Longitudinal sleep and mood charting; mania and depression scales; medication review	Diagnosis-specific pharmacotherapy; social-rhythm stabilization; relapse-prevention plan; CBT-I adapted to mood state
Developmentally sensitive phenotype	Nightmares, fear of sleeping alone, delayed sleep timing, digital sleep displacement, emotional dysregulation	Developmental changes in circadian timing and emotion regulation; family context; social-media exposure	Developmentally appropriate interview; parent/caregiver report; sleep diary; assessment of digital behavior	Family-based sleep intervention; digital hygiene; developmentally adapted CBT-I; treatment of trauma and emotional dysregulation

Note. This table is a conceptual synthesis, not an established clinical classification. The presentations are not mutually exclusive and may change over time or across affective states. Listed treatment targets are evidence-informed examples drawn from broader diagnostic or symptom-specific literatures and should not be interpreted as validated recommendations for the proposed presentation. Treatment should be individualized according to diagnosis, trauma history, developmental stage, comorbidity, medication exposure, patient preferences, and the strength of evidence. Key supporting evidence includes CBT-I across mental disorders (Hertenstein et al., 2022), IRT for post-trauma nightmares (Casement & Swanson, 2012; Prguda et al., 2023), and mixed evidence for prazosin in PTSD-related nightmares (Raskind et al., 2018). Abbreviations: CBT-I, cognitive behavioral therapy for insomnia; IRT, imagery rehearsal therapy; ISI, Insomnia Severity Index; PSG, polysomnography; PSQI, Pittsburgh Sleep Quality Index; PTSD, post-traumatic stress disorder; REM, rapid eye movement; STARSS, Seattle Trauma and Autonomic Reactivity Sleep Symptoms Scale.

## Data Availability

No new data were created or analyzed in this study.

## Abbreviations

The following abbreviations are used in this manuscript:

BD	bipolar disorder
BD-I	bipolar I disorder
BD-II	bipolar II disorder
BMI	body mass index
CBT	cognitive behavioral therapy
CBT-I	cognitive behavioral therapy for insomnia
CRP	C-reactive protein
CT	childhood trauma
DSM-5	Diagnostic and Statistical Manual of Mental Disorders, Fifth Edition
EA	emotional abuse
HPA	hypothalamic–pituitary–adrenal
IL-1β	interleukin-1 beta
IL-6	interleukin-6
IRT	imagery rehearsal therapy
MD	mood disorder
MDD	major depressive disorder
NREM	non-rapid eye movement
PA	physical abuse
PSG	polysomnography
PTSD	post-traumatic stress disorder
QoL	quality of life
REM	rapid eye movement
SA	sexual abuse
SSRI	selective serotonin reuptake inhibitor
STARSS	Seattle Trauma and Autonomic Reactivity Sleep Symptoms Scale
TA	adult trauma
